# Selective photocatalytic aerobic oxidation of methane into carbon monoxide over Ag/AgCl@SiO_2_†

**DOI:** 10.1039/d2sc01140a

**Published:** 2022-03-30

**Authors:** Jianxin Zhai, Baowen Zhou, Haihong Wu, Shuaiqiang Jia, Mengen Chu, Shitao Han, Wei Xia, Mingyuan He, Buxing Han

**Affiliations:** Shanghai Key Laboratory of Green Chemistry and Chemical Processes, School of Chemistry and Molecular Engineering, East China Normal University Shanghai 200062 China Mingyuanhe@126.com hhwu@chem.ecnu.edu.cn; Key Laboratory for Power Machinery and Engineering of Ministry of Education, School of Mechanical Engineering, Shanghai Jiao Tong University Shanghai 200240 China zhoubw@sjtu.edu.cn; Beijing National Laboratory for Molecular Sciences, CAS Key Laboratory of Colloid and Interface and Thermodynamics, CAS Research/Education Center for Excellence in Molecular Sciences, Institute of Chemistry, Chinese Academy of Sciences Beijing 100190 China hanbx@iccas.ac.cn

## Abstract

Design of active catalysts for chemical utilization of methane under mild conditions is of great importance, but remains a challenging task. Here, we prepared a Ag/AgCl with SiO_2_ coating (Ag/AgCl@SiO_2_) photocatalyst for methane oxidation to carbon monoxide. High carbon monoxide production (2.3 μmol h^−1^) and high selectivity (73%) were achieved. SiO_2_ plays a key role in the superior performance by increasing the lifetime of the photogenerated charge carriers. Based on a set of semi *in situ* infrared spectroscopy, electron paramagnetic resonance, and electronic property characterization studies, it is revealed that CH_4_ is effectively and selectively oxidized to CO by the *in situ* formation of singlet ^1^O_2_*via* the key intermediate of COOH*. Further study showed that the Ag/AgCl@SiO_2_ catalyst could also drive valuable conversion using real sunlight under ambient conditions. As far we know, this is the first work on the application of SiO_2_ modified Ag/AgCl in the methane oxidation reaction.

## Abstract 2

The Ag/AgCl@SiO_2_ catalyst exhibits excellent photocatalytic activity in selective aerobic oxidation of methane to carbon monoxide with high selectivity, and extended real light simulation feasibility shows potential in practical application.

## Graphical abstract

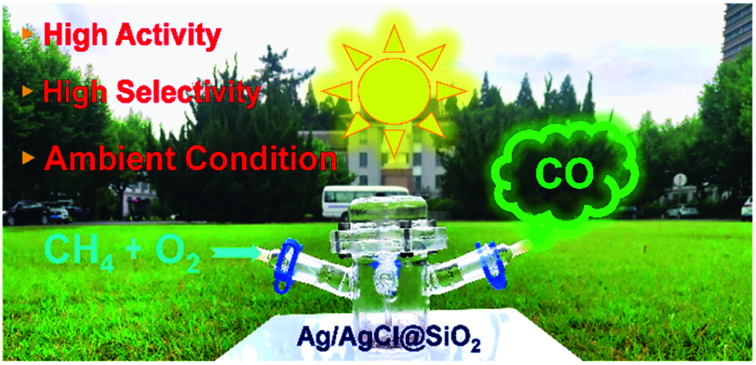

## Introduction

Highly efficient and selective transformation of CH_4_ is of great significance for the sustainable development of our society because of the revolution of shale gas.^1^ However, CH_4_ activation is a great challenge for catalysis because it is a very stable and inert molecule, and the unique symmetrical tetrahedral structure gives it four strong identical C–H bonds, which leads to a low electron and proton affinity, weak acidity, and low polarizability.^2^ Many efforts have been made on CH_4_ transformation by energy-intensive thermocatalytic routes.^3^ Carbon monoxide, which is a key component of syngas, is a valuable feedstock for manufacturing many products, such as alcohols, aldehydes, and hydrocarbon fuels. However, the mature industrial approach to producing syngas is mostly required to be done at high temperatures above 1000 K through the steam reforming of methane where high temperature is essential owing to the high C–H bonding energy (434 kJ mol^−1^) of the CH_4_ molecule.^4^ Hence selective oxidation of methane into carbon monoxide under ambient conditions paves a new avenue for syngas production but remains a grand challenge.

As a promising green strategy, photocatalytic CH_4_ oxidation can be carried out under mild conditions. The formation of an oxygen species which is reactive and electrophilic can initiate the dissociation of the C–H bond of CH_4_ at room temperature when excited by photons with several eV of energy.^5^ For this purpose, some materials have been used as photocatalysts in the gas or liquid phase, such as ZnO,^6^ WO_3_,^7^ SrTiO_3_,^8^ heteropolyacids,^9^ BiVO_4_,^10^ zeolite,^11^*etc.* However, low selectivity to target products, and often abundant CO_2_ production are still major problems. The activation energy of methane conversion is usually higher than that for products of high value-added C1 platform molecules such as carbon monoxide, methanol, formaldehyde *etc.*, resulting in the overoxidation of products and irreversible carbon loss. To date, research on photocatalytic conversion of CH_4_ to CO is relatively scarce.^12^

Among various photocatalysts, Ag@AgX (X = Cl, Br) possess excellent catalytic activity owing to the filled d^10^ electronic configuration of Ag^+^ ions, which can have a hand in the formation of the energy band structure or hybridization and strong absorptivity, which have attracted considerable attention.^13,14^ However, for a single component Ag@AgCl photocatalyst, the photogenerated electrons and holes easily recombine owing to the strong coulombic force between electrons and holes. It is known that the construction of hybrid heterostructures can effectively improve the photocatalytic performance. According to previous reports, as an emerging guest component, SiO_2_ has been used to build hybrid heterostructures for photocatalysis due to its low cost, excellent surface properties, UV-visible-IR optical transparency, and high stability/inertness.^15–17^ Based on the pioneering attempts, it is rational to improve the photocatalytic activity of Ag/AgCl by modification with SiO_2_.^18,19^

Herein, a method for preparing a Ag/AgCl@SiO_2_-*x* photocatalyst (*x* stands for wt% of SiO_2_ in the catalysts) is proposed, which used ionic liquid 1-octyl-3-methylimidazolium chloride ([Omim]Cl) as a Cl source. The catalysts were used in direct selective photocatalytic conversion of methane into carbon monoxide under ambient conditions for the first time. A series of characterization studies revealed that modification using SiO_2_ improved the photocatalytic activity. An appreciable carbon monoxide production of 2.3 μmol h^−1^, was achieved with a high selectivity of 73% under ambient conditions, presenting a green and viable route for the transformation of CH_4_ to carbon monoxide. In addition, the success in utilization of real sunlight to catalyse the valuable transformation indicated the potential for practical application.

## Results and discussion

**Fig. 1:**
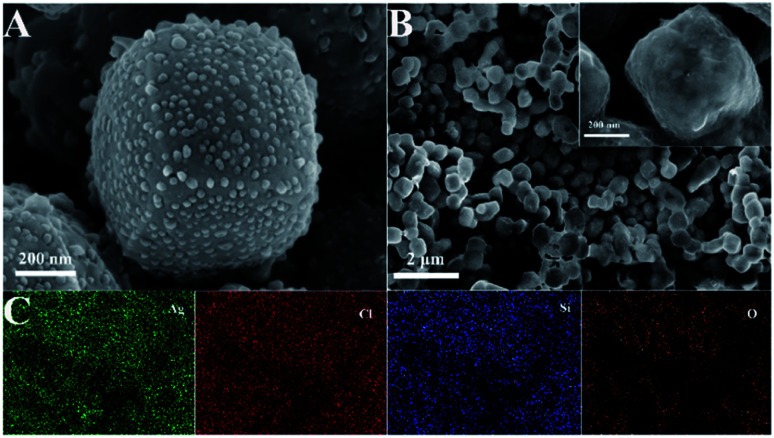
(A) SEM image of Ag/AgCl. (B) SEM image of Ag/AgCl@SiO_2_-4.1%. (C) The elemental mapping of Ag/AgCl@SiO_2_-4.1%.

Scanning electron microscopy (SEM) characterization studies were carried out to show the morphology and microstructure of Ag/AgCl and Ag/AgCl@SiO_2_-4.1%, as shown in Fig. 1. It can be seen that Ag/AgCl had a cubic morphology with a uniform size and its surface was decorated with small particles. The width of the cubes was about 400 nm as shown in Fig. 1A. The small particles on the surface were identified as Ag particles.^20^ The small particles could not be observed on the surface of Ag/AgCl@SiO_2_-4.1% (Fig. 1B), indicating that the Ag/AgCl cube was covered by SiO_2_. According to the different colour contrasts of the pictures in Fig. S1,† the element mappings of Cl and Ag of Ag/AgCl can be clearly observed. Ag, Cl, Si and O are uniformly distributed in Ag/AgCl@SiO_2_-4.1% in Fig. 1C and S2.† These results further demonstrated that Ag/AgCl@SiO_2_ was prepared. In this design, SiO_2_ can function as a blocking layer of the Ag/AgCl photocatalyst for suppressing electron–hole pair recombination. Moreover, the high surface area of SiO_2_ is beneficial for reactant adsorption. Together, the incorporated SiO_2_ promotes the reaction, which will be discussed in the following sections.

**Fig. 2:**
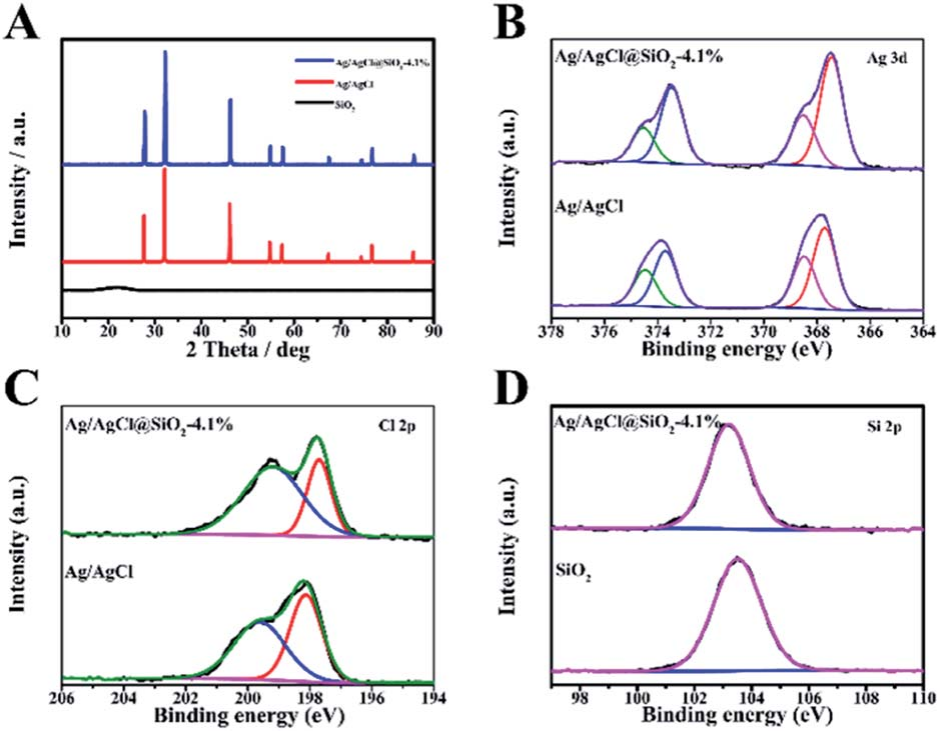
(A) X-ray diffraction (XRD) patterns for Ag/AgCl, SiO_2_ and Ag/AgCl@SiO_2_-4.1%. (B) Ag 3d X-ray photoelectron spectroscopy (XPS) spectra of Ag/AgCl and Ag/AgCl@SiO_2_-4.1%. (C) Cl 2p XPS spectra of Ag/AgCl and Ag/AgCl@SiO_2_-4.1%. (D) Si 2p XPS spectra of SiO_2_ and Ag/AgCl@SiO_2_-4.1%.

The crystalline structure and composition of the samples with varied proportion of SiO_2_ were analysed by the X-ray diffraction (XRD) technique (Fig. 2A and S3†). The broad peak observed at 21.6 degrees corresponds to the partially crystalline SiO_2_. The featured peaks of Ag/AgCl at 27.8, 32.2, 46.2, 54.8, 57.8, 67.4, 74.5,76.7 and 85.7 are well consistent with the typical (111), (200), (220), (311), (222), (400), (331), (420) and (422) planes of AgCl (JCPDS number 31-1238). For the Ag/AgCl and Ag/AgCl@SiO_2_ samples, similar reflections were identified, indicating that the introduction of SiO_2_ had no obvious influence on their crystalline structure (Fig. S3†).

The XPS method was also used to study the composite materials. The XPS survey spectra of Ag/AgCl@SiO_2_-4.1% are presented in Fig. S4.† In addition, the high-resolution spectra provide the binding energies of Ag 3d in Fig. 2B. The binding energies of 367.5 eV and 373.5 eV correspond to 3d_5/2_ and 3d_3/2_ of Ag^+^ and 368.5 eV and 375.6 eV correspond to 3d_5/2_ and 3d_3/2_ of Ag^0^ respectively. The spectra reveal that a considerable amount of Ag exists in the metallic form.^21^ In Fig. 2C, the peaks of Cl 2p_3/2_ and Cl 2p_1/2_ with binding energies of 197.7 and 199.2 eV indicate the presence of Cl^−^.^22^ In the region of Si 2p (Fig. 2D), the binding energy of Ag/AgCl@SiO_2_-4.1% was 103.2 eV, corresponding to the Si–O–Si bond.^23^ As a whole, the XPS data further confirmed the successful synthesis of Ag/AgCl@SiO_2_ hybrid heterostructures, which agreed well with the SEM and XRD results.

**Fig. 3:**
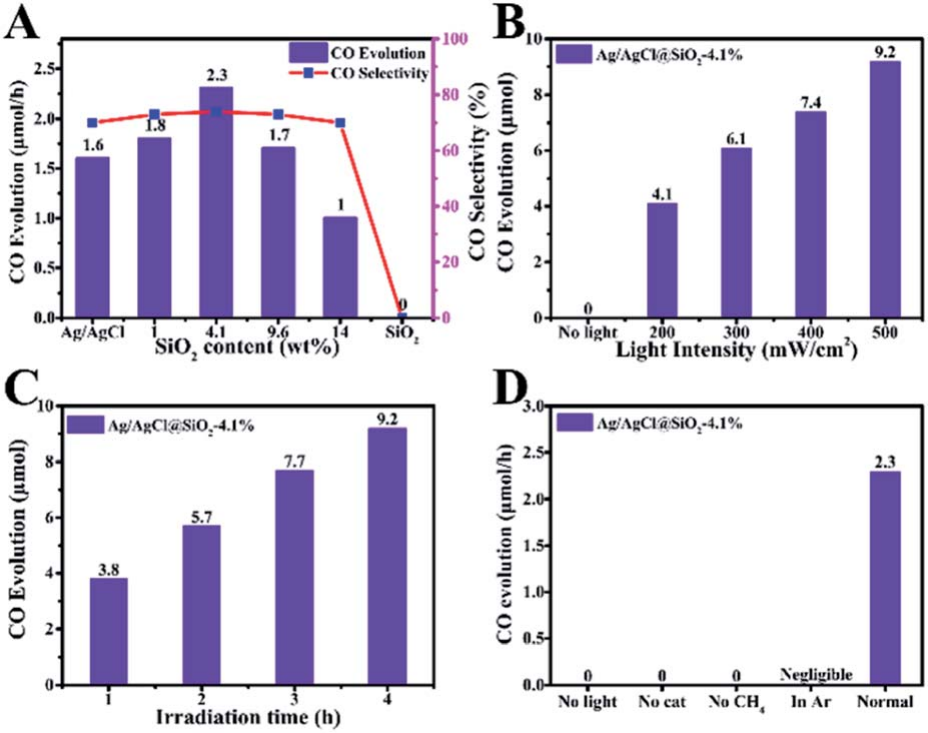
(A) Influence of the SiO_2_ content in Ag/AgCl@SiO_2_-*x*. (B) Influence of light intensity on CO evolution over Ag/AgCl@SiO_2_-4.1%. (C) CO evolution of the photocatalyst Ag/AgCl@SiO_2_-4.1% at different reaction times. (D) Control experiments in the absence of light, the Ag/AgCl@SiO_2_-4.1% photocatalyst, CH_4_ and CH_4_/O_2_. The amount of every catalyst used in the photoreaction is 10 mg.

We next studied the activity and selectivity of Ag/AgCl @SiO_2_-*x* for the oxidation of CH_4_ to CO. Fig. 3A shows the product distribution after 4 hours of light irradiation. CO was dominant with CO_2_ and hydrogen as the by-products. Apart from the gas products, the possible liquid products were analyzed by nuclear magnetic resonance (NMR) spectroscopy. It was found that no liquid products were produced from the photocatalytic aerobic oxidation of methane. It is clear that the introduction of SiO_2_ can dramatically enhance the photocatalytic performance of the catalysts for methane conversion. In comparison with Ag/AgCl, the CO production rate is enhanced by SiO_2_, and Ag/AgCl@SiO_2_-4.1% yields the highest conversion rate of 2.3 μmol h^−1^ and a CO selectivity of 73%, which is nearly 1.4 times that of Ag/AgCl (1.6 μmol h^−1^). In this case, there is an optimal balance among electron–hole pair recombination, catalytic sites-reactant interaction and light collection, thus leading to an optimum activity. Further increasing the loading amount of SiO_2_ does not increase CH_4_ conversion. This may be because as too much SiO_2_ was added, the effect of the decreasing photoactive component Ag/AgCl became a dominant factor, thus resulting in the lower activity.

The performance of the Ag/AgCl@SiO_2_-4.1% catalyst at different light intensities was evaluated and the results are shown in Fig. 3B. The rate of CO generation increases with increasing light intensity, which indicates that light was the driving force of the transformation, and Ag/AgCl@SiO_2_-4.1% was not ruined under light illumination. Apart from the outstanding photocatalytic activity, the stability of the photocatalyst is another important aspect to be considered. Fig. 3C shows the time-course CO evolution at different times. The yield increased continuously with time, suggesting good stability of the catalyst. The control experiments showed that the carbonous product could not be generated in the absence of the Ag/AgCl@SiO_2_-4.1% photocatalyst or light, indicating that the photocatalyst and light irradiation are necessary for CH_4_ conversion (Fig. 3D). Furthermore, no carbonous products were detected under an Ar atmosphere or without CH_4_, confirming that CO is produced form CH_4_. In addition, the relationship between CO evolution and the light wavelength was studied. As shown in Fig. S5,† the formation trend of CO was well consistent with the light absorption trend of the Ag/AgCl@SiO_2_-4.1% photocatalyst, and the apparent quantum efficiency (AQE) is determined to be 0.26% at 313 nm. The results further validated that the reaction proceeded *via* photocatalysis. The photoexcitation of the Ag/AgCl@SiO_2_-4.1% photocatalyst by photons with energy higher than its bandgap is the first step of methane oxidation, which can initiate the subsequent reactions.

The N_2_ adsorption–desorption isotherms of Ag/AgCl@SiO_2_-*x* were measured (Fig. S6†). It is shown that the BET surface areas for Ag/AgCl@SiO_2_-*x* composites increased with the content of SiO_2_. The surface area of Ag/AgCl@SiO_2_-4.1% (3.52 m^2^ g^−1^) was nearly three times higher than that of Ag/AgCl (0.872 m^2^ g^−1^). The larger surface area resulted from the porous SiO_2_ layer.

**Fig. 4:**
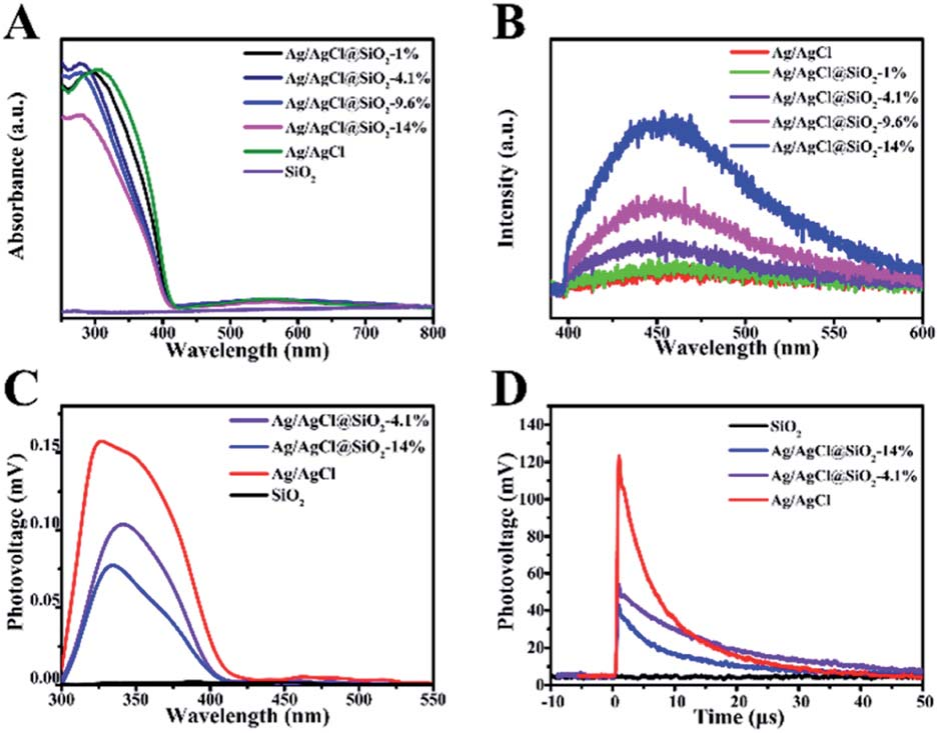
(A) UV-vis absorption spectra of Ag/AgCl@SiO_2_-*x* catalysts. (B) Room temperature photoluminescence (PL) spectra of Ag/AgCl@SiO_2_-*x*. (C) Surface photovoltage response of Ag/AgCl@SiO_2_-*x*. (D) TPV of Ag/AgCl@SiO_2_-*x*.

UV-vis diffuse reflectance spectra of Ag/AgCl@SiO_2_ were obtained to explore the influence of SiO_2_ on the optical properties of the samples (Fig. 4A).^24^ The absorption band of pure AgCl was in the region of 250–400 nm. In the case of the Ag/AgCl composite, two prominent absorption bands are observed in the UV-visible region. The former can be attributed to the absorption of AgCl, and its corresponding absorption edge was located below 400 nm. The latter absorption bands (*λ*_max_ 550 nm) can be credited to the characteristic absorption of the local surface plasmon resonance of the metallic silver covering on the AgCl surface. Furthermore, it is clearly seen that light harvesting decreased with the increasing content of SiO_2_, duo to the decreasing content of photoactive Ag/AgCl in Ag/AgCl@SiO_2_-*x*. Photoluminescence (PL) was utilized to elucidate the recombination of the photoexcited electrons and holes within the Ag/AgCl@SiO_2_-*x* composites. In Fig. 4B, the PL spectra of both Ag/AgCl and Ag/AgCl@SiO_2_-*x* exhibited broad bands, suggesting multiple radiation processes of the excited charge carriers. With an increasing silica content, the wider, stronger peak of Ag/AgCl@SiO_2_-*x* became more obvious, suggesting that more carriers were produced.^25,26^

Electronic signal change on the surface of the prepared Ag/AgCl@SiO_2_-*x* samples was further studied by using surface photovoltage (SPV) spectra, and the results are presented in Fig. 4C. It is shown that SiO_2_ could not be photoexcited in the ultraviolet-visible region ranging from 300 to 550 nm, while an approximately 0.15 mV photovoltage was observed for Ag/AgCl in 300 nm to 400 nm region. This is because Ag/AgCl can effectively absorb light in this region. After coating with SiO_2_, the Ag/AgCl@SiO_2_-*x* photovoltage is also observed in the region of 300–400 nm, and the photovoltage decreased with the increase of the content of the silica. Therefore, the weak surface net charges and SPV response on Ag/AgCl@SiO_2_-*x* indicate that the extraction of photogenerated electrons was not significantly blocked and suppressed the recombination of photogenerated charges, which favours CH_4_ oxidation by the photoexcited charges.^27^

To further explore the photogenerated charge-transfer behaviour, the surface transient photovoltage (TPV) was measured. In Fig. 4D, at times longer than 0.7 μs, the TPV signal of Ag/AgCl decreased at a fast rate while the response signal of Ag/AgCl@SiO_2_-*x* decreased slowly. Moreover, the longer decay lifetime (8.93 μs) of Ag/AgCl@SiO_2_-4.1% in comparison with that of Ag/AgCl (5.95 μs), reveals the enhanced lifetime of charge carriers of Ag/AgCl by the coating of SiO_2_.^28–30^ The longer lifetime of the photogenerated charges is favourable to the subsequent photocatalytic CH_4_ oxidation reaction. This may be a key factor for the improved photocatalytic activity of the Ag/AgCl particles after the incorporation of SiO_2_.^31^

**Fig. 5:**
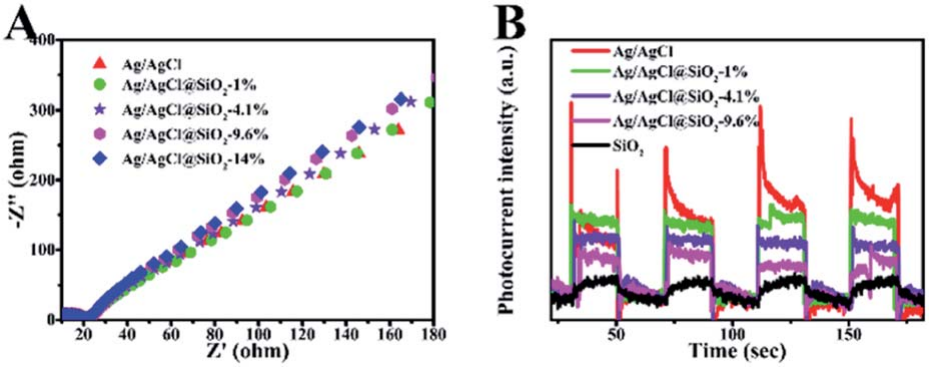
(A) Electrochemical impedance spectra (EIS) of Ag/AgCl@SiO_2_-*x*. (B) Periodic on/off photocurrent response of Ag/AgCl@SiO_2_-*x*.

To evaluate the kinetics of charge transfer in the heterostructures, the electrochemical impedance spectroscopy (EIS) measurements and transient photocurrent response analysis were further conducted. As shown in Fig. 5A, all the samples examined showed similar Nyquist plots, suggesting that the SiO_2_ coating did not significantly increase the resistance of charge carrier migration in the composite while suppressing their recombination as suggested by the TPV measurement above. Fig. 5B shows the transient photocurrent response of the Ag/AgCl and Ag/AgCl@SiO_2_-*x* electrodes over several on–off cycles of intermittent light irradiation. It is shown that SiO_2_ reduced the transient photocurrent response, and the effect was more obvious at a higher silica content. This was mainly because the addition of a SiO_2_ layer reduced the content of Ag/AgCl in Ag/AgCl@SiO_2_-*x* as verified by the optical property characterization in Fig. 4A.^23,32^ The combination of the photoelectrochemical property characterization studies with photocatalytic performance indicated that although the incorporated SiO_2_ will affect the active sites more or less, Ag/AgCl@SiO_2_-4.1% demonstrated an optimal balance for light absorption, electron–hole pair recombination, as well as catalytic sites-reactant interaction. As a result, an optimal CO evolution rate of 2.3 μmol h^−1^ could be achieved from methane oxidation with 73% selectivity.

**Fig. 6:**
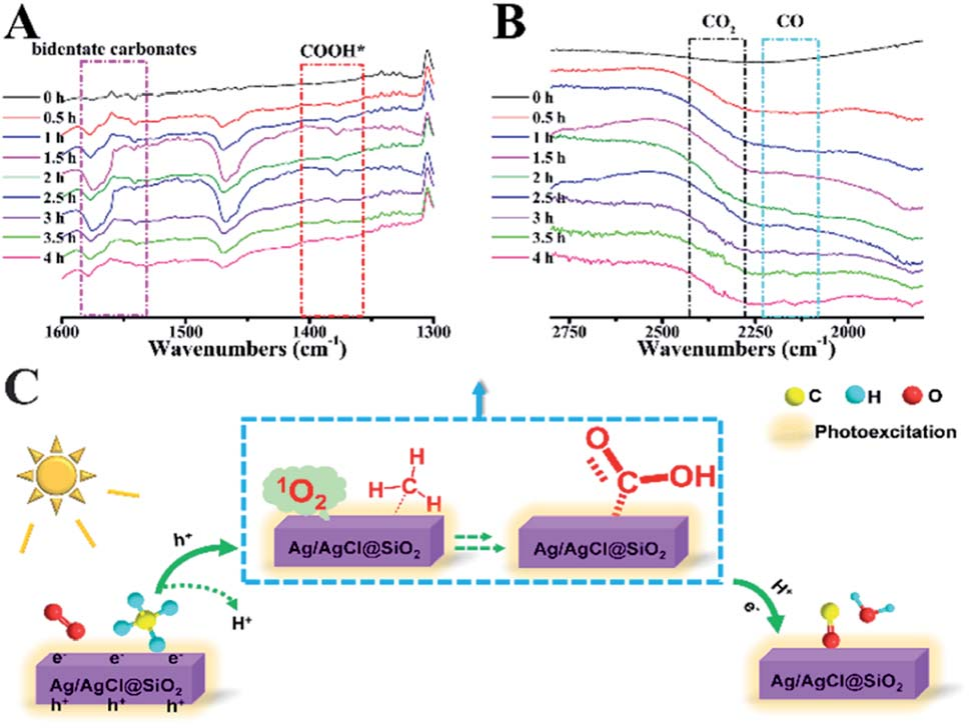
(A and B) FTIR spectra of the gaseous phase during methane photocatalytic oxidation over the Ag/AgCl@SiO_2_-4.1% catalyst as a function of reaction time. (C) The possible mechanism of CH_4_ oxidation over Ag/AgCl@SiO_2_ under light illumination.

A semi *in situ* IR analysis was performed to gain insight into the reaction mechanism. In Fig. 6A, the IR band at 1307 cm^−1^ is assigned to the typical C–H rotation-bending of the absorbed saturated –CH_*x*_ on the Ag/AgCl@SiO_2_-4.1% surface.^33^ Upon light illumination, a new broad peak at 1384 cm^−1^ emerged, which is attributable to the adsorbed formate (COOH*) species (Fig. 6A).^34,35^ The peaks at 1520–1580 cm^−1^ primarily originated from the bidentate carbonates bound to the catalyst surface, further validating the formation of a COOH* intermediate during the process.^4,9^ Significantly, gaseous carbon monoxide was identified by the rotation-stretching bands at 2130 cm^−1^, which was enhanced by increasing the illumination time (Fig. 6B). Meanwhile, the featured band at 1650 cm^−1^ credited to H_2_O was also observed at different illumination times as well (Fig. S8†), which is associated with COOH* dehydration as suggested by previous studies.^35^ The bands assigned to the characteristic mode of CO_2_ at 2330–2370 cm^−1^ gradually increased arising from the unavoidable overoxidation, well explaining the fact that the highest selectivity of CO was 73%.^36^ In the cycle, the critical role of O_2_ was studied by *in situ* electron paramagnetic resonance (EPR). As shown in Fig. S9,† upon illumination, singlet oxygen (^1^O_2_) was produced. The *in situ* formed singlet oxygen is rationally considered to play an important role in CH_4_ oxidation because of its unique oxidizing capability. Based on the above results, the possible mechanism of photocatalytic oxidation of CH_4_ is proposed (Fig. 6C). Methane molecules are first adsorbed on the Ag/AgCl@SiO_2_ surface. When illuminated, CH_4_ would be oxidized by the photogenerated holes to release H^+^. Meanwhile, Ag/AgCl@SiO_2_ in the photoexcited state would transfer energy to the absorbed O_2_ molecule in the ground state for producing singlet oxygen(^1^O_2_).^37–40^ The *in situ* formed singlet ^1^O_2_ will be subsequently involved in the activation of the C–H bonds to form the key intermediate of COOH*.^35^ Eventually, CO is formed from COOH* dehydration in the presence of photoinduced electrons and protons,^41^ concurrent with the formation of H_2_O, as characterized by *in situ* IR characterization (Fig. S8†) and previous discoveries.^37–40^

**Fig. 7:**
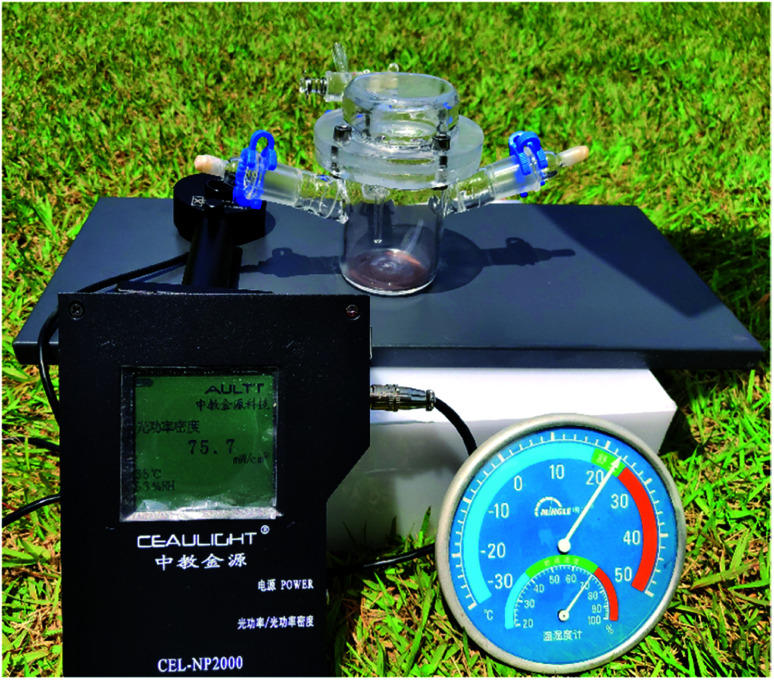
Photograph of the outdoor experimental test setup.

Utilization of solar energy directly is more interesting from a practical application point of view. Therefore, an outdoor solar-driven test was conducted under ambient conditions from 10:30 to 14:30 at East China Normal University, Shanghai (latitude 31°232732′ N and longitude 121°411581′ E) (Fig. 7). 10 mg of the Ag/AgCl@SiO_2_-4.1% photocatalyst was placed in a quartz reactor (250 mL) with an irradiation area of 19.6 cm^2^ and filled with mixture gas (0.2% CH_4_/1% O_2_/98.8% Ar) prior to illumination. The average CO evolution rate reached a value of 1.2 μmol h^−1^ under real solar illumination at 75.7 mW cm^−2^. Ag/AgCl@SiO_2_-4.1% was also validated to be a robust catalyst during the 4 hour testing period. The results above suggest the viability of the designed Ag/AgCl@SiO_2_ catalyst for photocatalytic methane oxidation powered by solar energy under ambient conditions. For the separation problems that may be encountered in future industrial applications, we believe that membrane separation is one of the most suitable technologies because the mixture consisted mainly of CO, CO_2_, and CH_4_.

## Conclusions

To summarize, Ag/AgCl@SiO_2_ photocatalysts have been prepared for CH_4_ oxidation to CO under ambient conditions. The decorated SiO_2_ coating plays a key role in enhancing the activity and selectivity of CO by balancing among electron–hole pair recombination, catalytic sites-reactant interaction, and light collection and absorption. Under the optimized conditions, an appreciable activity of 2.3 μmol h^−1^ can be achieved for CO evolution with a high selectivity of 73%. A mechanism is proposed, revealing that CO is evolved from CH_4_ oxidation *via* the key intermediate of COOH* by using the *in situ* singlet ^1^O_2_ yielded over photoexcited Ag/AgCl–SiO_2_. In addition, outdoor testing further indicates that photocatalytic CH_4_ oxidation toward CO can be carried out efficiently using real sunlight under ambient conditions, showing the potential for application.

## Experimental

### Chemicals

Silver nitrate (AgNO_3_) was purchased from Sinopharm Chemical Reagent Co., Ltd. Tetraethyl orthosilicate (TEOS) was obtained from Aladdin Industrial Co., Ltd. Ionic liquid 1-octyl-3-methylimidazolium chloride ([Omim]Cl) was provided by Shanghai Cheng Jie Chemical Co., Ltd. All the chemicals were used directly without further treatment. Deionized water was used for all of the experiments.

### Materials synthesis

1 mmol AgNO_3_ and 2 mmol [Omim]Cl were dissolved in water (10 mL) rapidly with vigorous stirring for 0.5 h (labelled as solution A). Then a certain amount of TEOS (0.05 mL to 0.2 mL) was added to water (10 mL) and stirred for 0.5 h (labelled as solution B). 0.1 mL NaOH (1 M) was dropped into solution A. Then solution B was added into solution A drop by drop under stirring. The mixture was magnetically stirred at room temperature for 24 h. The obtained precipitate was thoroughly washed with DI water and vacuum freeze dried overnight.

### Photocatalytic tests

The photocatalytic conversion of methane was carried out in a glass reactor (250 mL) with a quartz window under atmospheric pressure. A 300 W Xe lamp (Beijing China Education Au-Light Co., Ltd) equipped with a 300–800 nm cut-off filter was used as the light source to get light of the desired wavelength (300–800 nm). In a typical process, 10 mg of the catalyst powder was dispersed in 10 mL water in a reactor, and the reactor was heated at 333 K overnight to volatilize the solvent and a thin film of the catalyst was formed for illumination reaction. Prior to the illumination reaction, this reactor was evacuated by using a vacuum pump, and then filled with the mixture gas (0.2% CH_4_/1% O_2_/98.8% Ar) at atmospheric pressure. This evacuation-filling operation was repeated three times. During the whole experiment, the reactor was wrapped in aluminium foil to avoid light interference from the surroundings and the temperature was controlled by using a water bath set at 298 K. After a desired reaction time, the gas products were detected by using a gas chromatograph (Agilent GC-8860) and calibrated with a standard gas mixture. After the reaction, 10 mL of distilled water was injected into the reaction chamber; and the suspended photocatalyst was removed by filtration. The liquid mixture was subsequently characterized by using a nuclear magnetic resonance spectrometer (Bruker Avance III HD 500).

### Materials characterization

The morphology of the samples was characterized by using a Zeiss Sigma HD scanning electron microscope (SEM). A Rigaku Ultima VI X-ray diffractometer was used to record the X-ray diffraction patterns with a scanning speed of 5° min^−1^ between 10° and 90°, which was operated at 25 kV and 35 mA with Cu Kα radiation. X-ray photoelectron spectroscopy (XPS) data were obtained on an AXIS Supra surface analysis instrument using a monochromatic Al Kα X-ray beam (1486.6 eV). BET measurements were carried out using N_2_ at −196 °C in Quadrasorb evo equipment. UV-vis diffuse reflectance spectra were obtained by using a UV-Visible spectrophotometer with a diffuse reflectance unit, (UV-2700, Shimadzu, Japan) where BaSO_4_ was used as the internal reflectance standard. A liquid nitrogen cooled charge coupled device (CCD) spectrometer (Princeton Instruments) was used to detect the steady-state PL spectra under 375 nm excitation. The surface photovoltage measurements were carried out on a surface photocurrent spectroscope (CEL-SPS1000). The transient-state surface photovoltage measurements were carried out on a CEL-TPV2000 device. Semi *in situ* FTIR spectra were recorded with a NICOLET iS50 FTIR spectrometer (Thermo SCIENTIFIC, USA) equipped with a high-temperature reaction chamber and a mercury cadmium telluride (MCT) detector at a resolution of 4 cm^−1^ and 32 scans per spectrum. The background spectrum was scanned before the mixture gas (0.2% CH_4_/1% O_2_/98.8% Ar) was introduced. The Si contents were quantified by using an inductively coupled plasma emission spectrometer (ICP-OES) on an Optima 8300. The *in situ* electron paramagnetic resonance (EPR) measurement was carried out using an EMXplus-10/12 to detect ^1^O_2_ radicals by adding 2,2,6,6-tetramethylpiperidine (TEMP) as a spin-trapping reagent in the photocatalytic reaction after the mixture gas (75% CH_4_/25% O_2_) was introduced. A 300 W Xe lamp (Beijing China Education Au-Light Co., Ltd) equipped with a 300–800 nm cut-off filter was used as the light source to get light of the desired wavelength (300–800 nm).

### The photoelectrochemical (PEC) tests

The photoelectrochemical tests of the samples were carried out on an electrochemical workstation (CHI660E, Chenhua Instrument, Shanghai, China) by using a three-electrode system. The catalyst was drop-coated on clean FTO glass, which was used as the working electrode, while Pt and Ag/AgCl electrodes acted as the counter and reference electrodes, respectively. A 300 W Xe lamp (Aulight, Beijing) acted as the light source and all of the electrochemical tests were carried out in 0.1 mol L^−1^ sodium sulfate solution.

### Apparent quantum yield (AQY) calculation

The AQY for CO evolution for Ag/AgCl@SiO_2_-*x* was measured using a standard experimental setup. The system was irradiated by using a 300 W Xe lamp equipped with a 313 nm optical band-pass filter and subjected to reaction for 4 h. The average intensity of irradiation (*I*) was determined by using a CEL-NP2000 spectrometer. The irradiation area (*S*) was measured to be 19.63 cm^2^. *E*_*λ*_ is given by *hc*/*λ* (*λ* = 313 nm). The AQY was calculated by using the following equation.
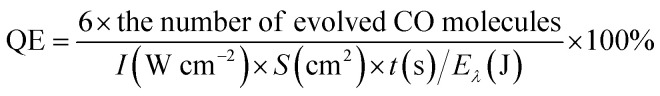


## Data availability

The authors declare that all data supporting the findings of this study are available within the paper [and its ESI].†

## Author contributions

J. X. Zhai, B. W. Zhou and H. H. Wu conceived the idea. J. X. Zhai, S. Q. Jia, M. E. Chu, S. T. Han and W. Xia carried out the sample synthesis, characterization and photochemical test. B. W. Zhou, H. H. Wu, M. Y. He and B. X. Han were responsible for the overall direction of the project. All the authors contributed to the overall scientific interpretation and edited the manuscript.

## Conflicts of interest

The authors declare no competing interests.

## Supplementary Material

SC-013-D2SC01140A-s001
