# Supplementary material for: Selective photocatalytic aerobic oxidation of methane into carbon monoxide over Ag/AgCl@SiO2
Source: Chem Sci. 2022 Mar 30;13(16):4616–22. doi: 10.1039/d2sc01140a (PMC9020177; doi:10.1039/d2sc01140a)
Supplement: SC-013-D2SC01140A-s001 [file SC-013-D2SC01140A-s001.pdf]

Electronic Supplementary Material (ESI) for Chemical Science.
This journal is © The Royal Society of Chemistry 2022

Supplementary Materials for

Selective photocatalytic aerobic oxidation of methane into carbon monoxide over $Ag/AgCl@SiO_2$

Jianxin Zhai, Baowen Zhou,* Haihong Wu,* Shuaiqiang Jia, Mengen Chu, Shitao Han, Wei Xia, Mingyuan He,* Buxing Han*

*Corresponding author. Email: zhoubw@sjtu.edu.cn (B.W.Z.); hhwu@chem.ecnu.edu.cn (H.H.W.); Mingyuanhe@126.com (M.Y.H.); hanbx@iccas.ac.cn (B.X.H.).

This file includes:

Fig. S1. The elemental mapping of Ag/AgCl.
Fig. S2. EDS analysis of Ag/AgCl and Ag/AgCl@SiO_2-4.1%.
Fig. S3. XRD patterns for Ag/AgCl@SiO_2-x.
Fig. S4. XPS survey spectra of Ag/AgCl@SiO_2-4.1%.
Fig. S5. Light wavelength dependence of yields of CO on the light absorption spectrum of Ag/AgCl@SiO_2-4.1% photocatalyst.
Fig. S6. N_2 adsorption-desorption isotherms of Ag/AgCl@SiO_2-x.
Fig. S7. Photograph of the semi in-situ IR analysis apparatus.
Fig. S8. FTIR spectra of gaseous phase during methane photocatalytic oxidation over the Ag/AgCl@SiO_2-4.1% catalyst as a function of reaction time.
Fig. S9. In situ ESR spectra of Ag/AgCl@SiO_2-4.1% in the presence of TEMP.
Table S1.ICP－OES analysis of Ag/AgCl@SiO_2-x.

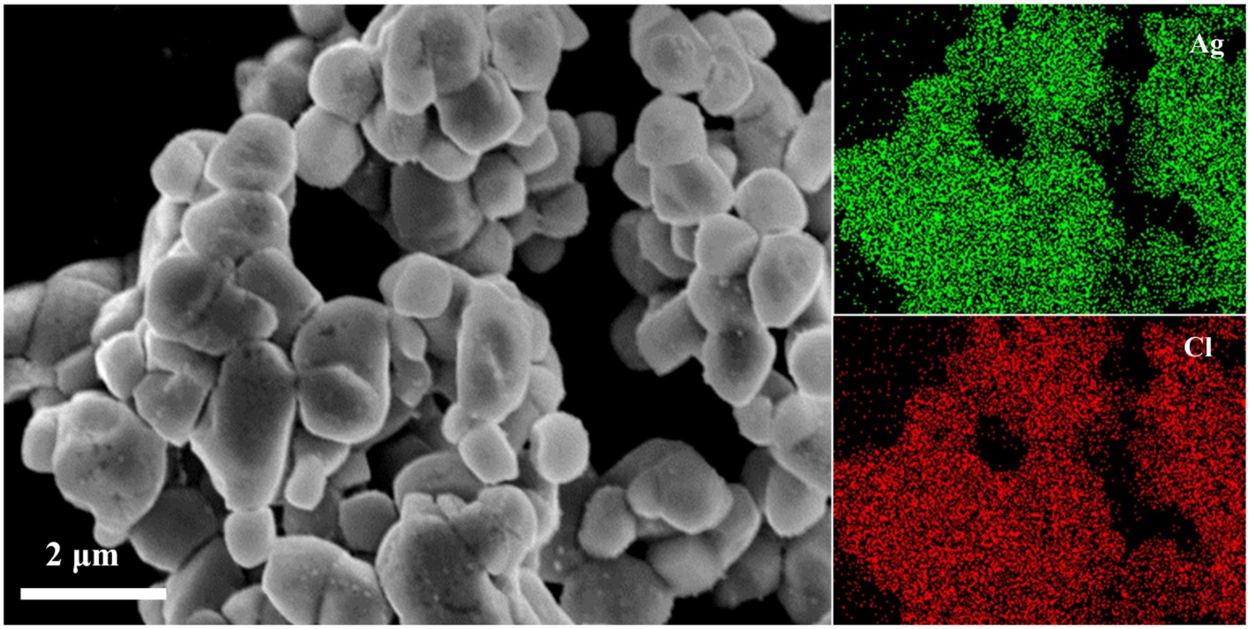

Fig. S1. The elemental mapping of Ag/AgCl.

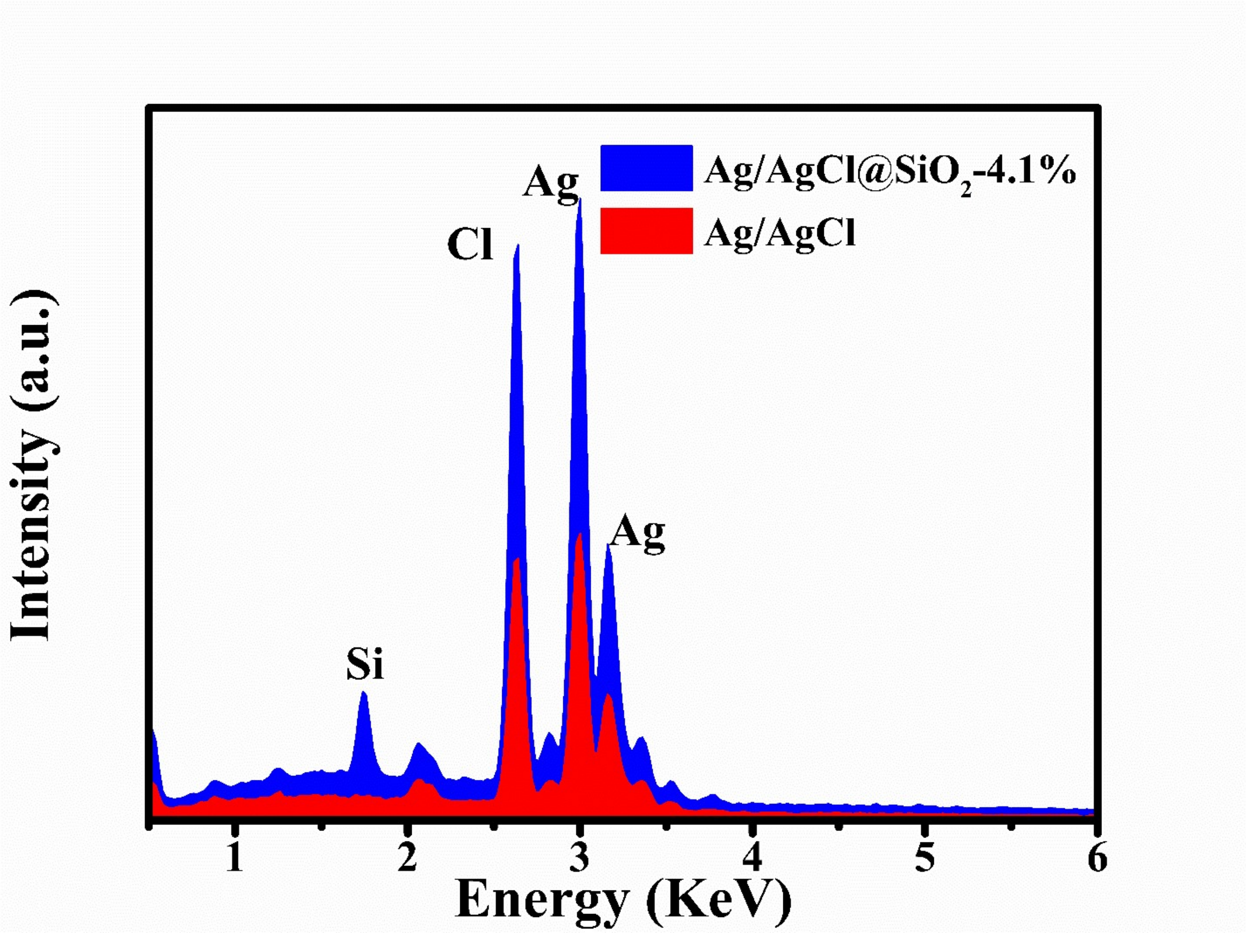

Fig. S2. EDS analysis of Ag/AgCl and Ag/AgCl@SiO_2-4.1%.

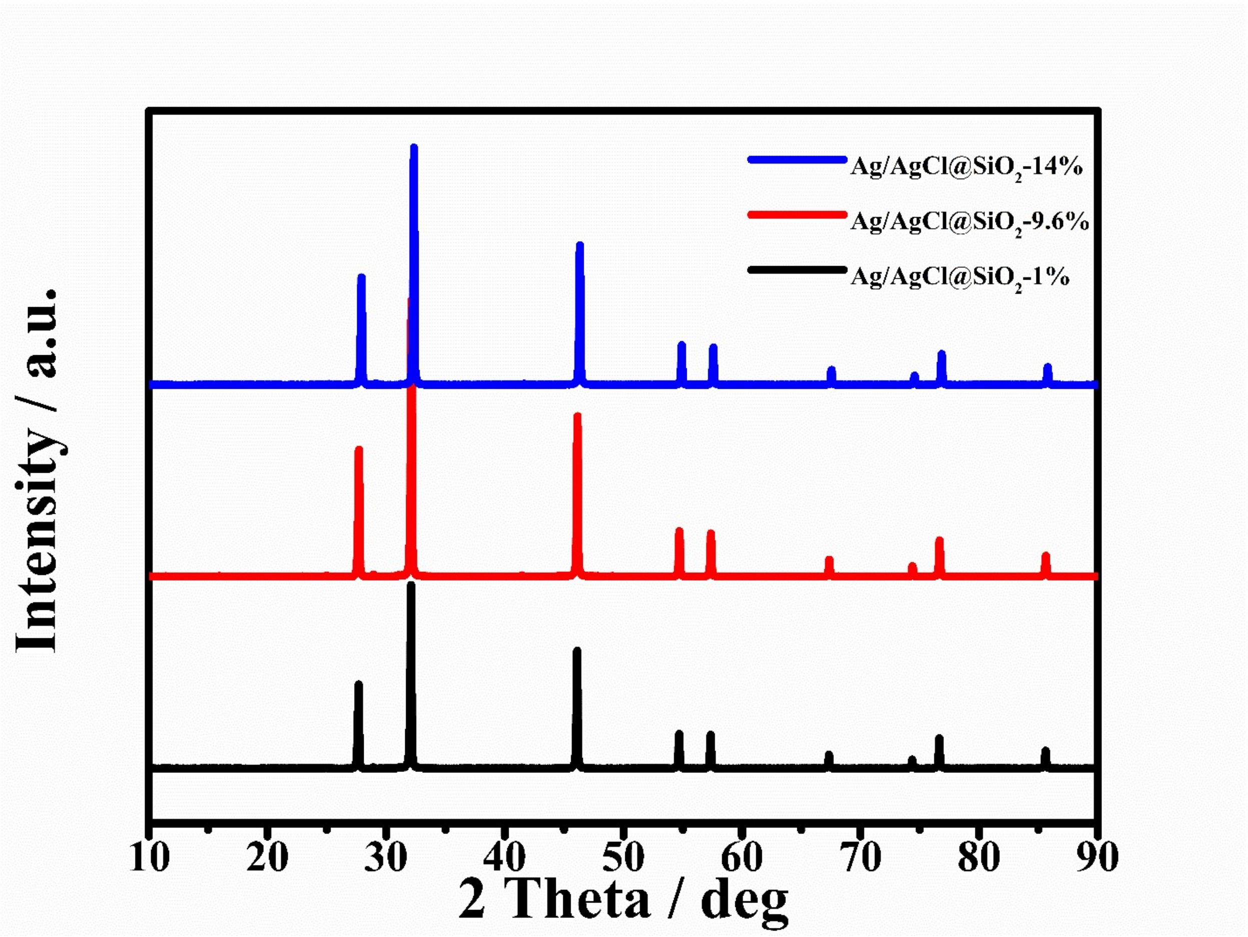

Fig. S3. XRD patterns for Ag/AgCl@SiO_2-x.

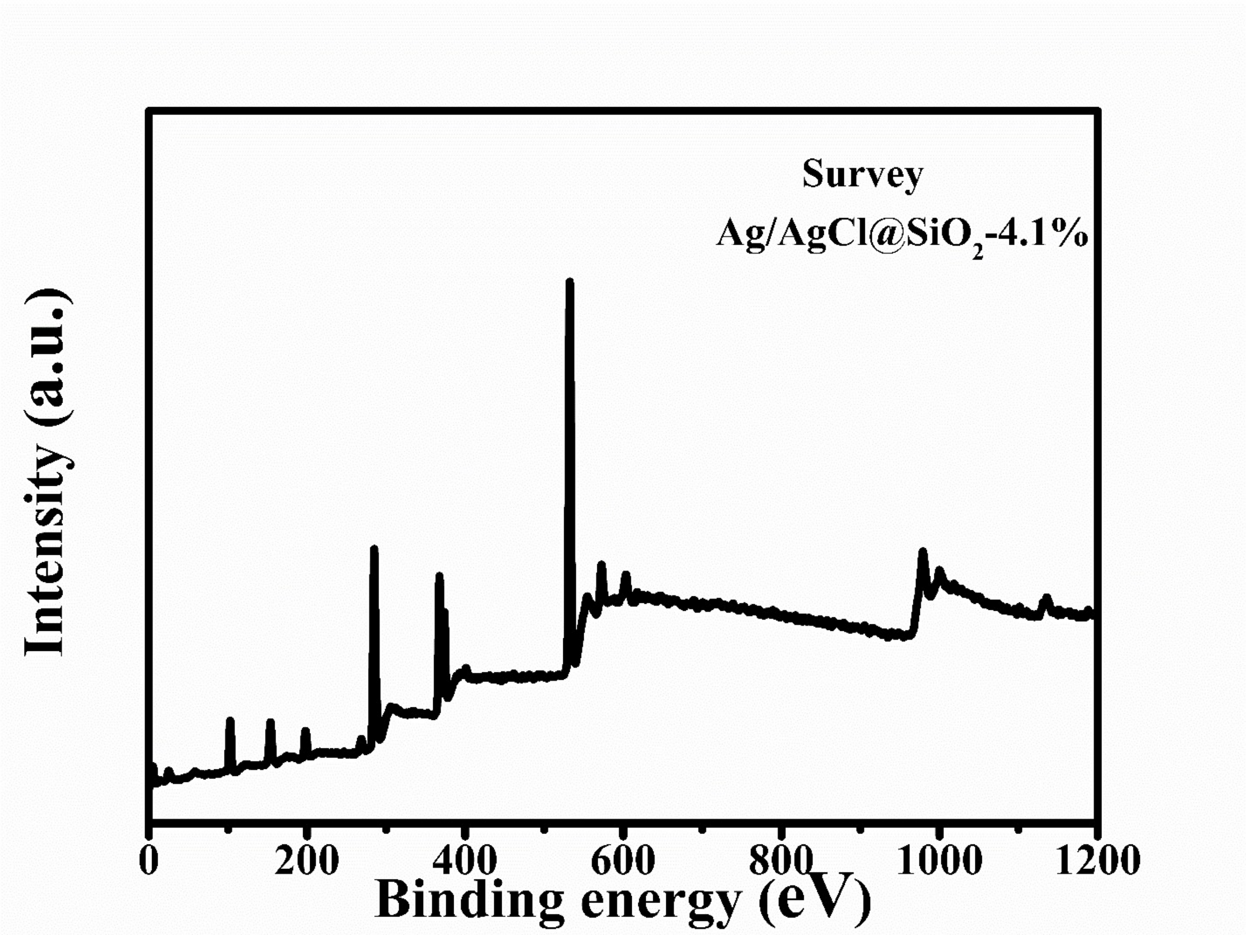

Fig. S4. XPS survey spectrum of Ag/AgCl@SiO_2-4.1%.

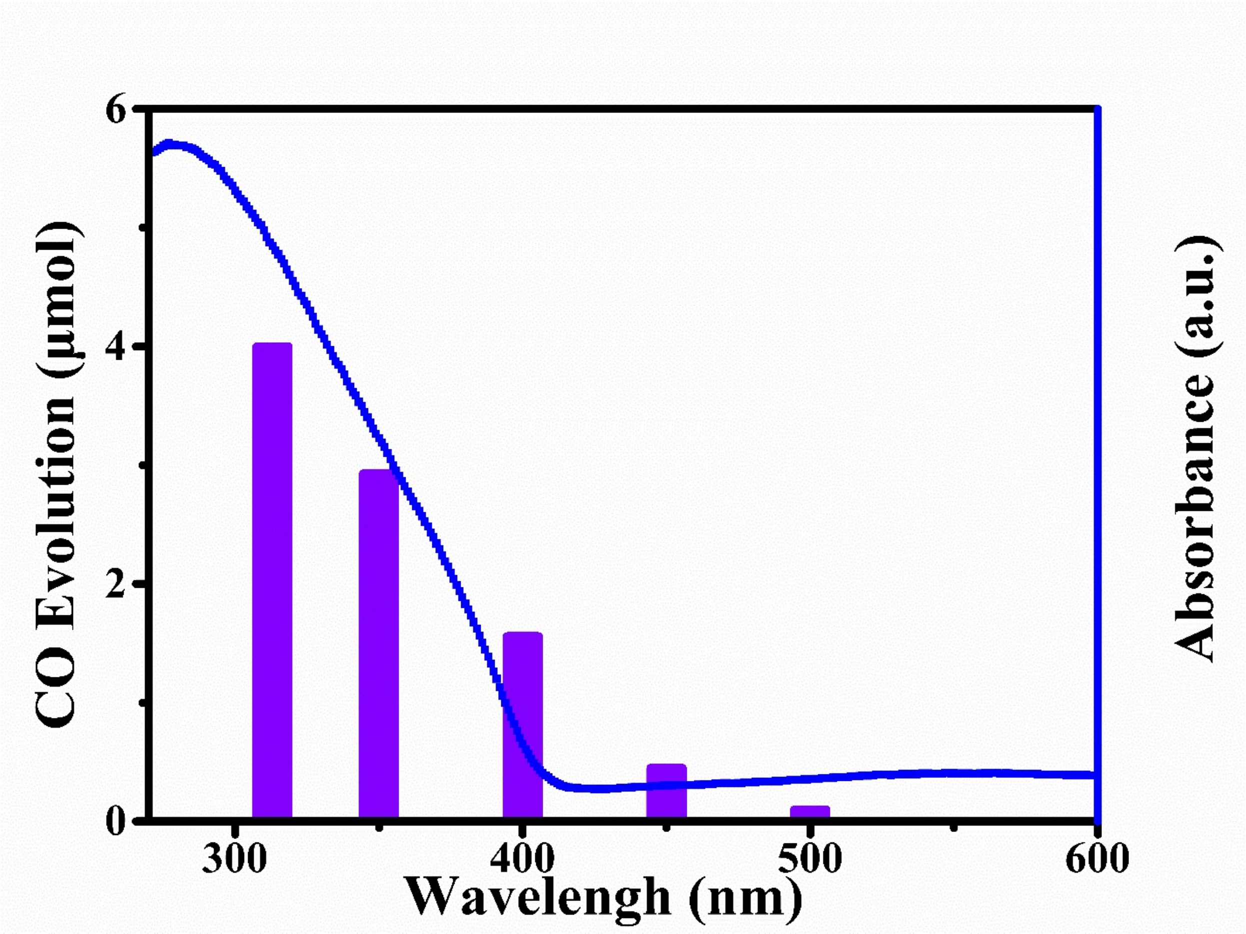

Fig. S5. Light wavelength dependence of yields of CO on the light absorption spectrum of Ag/AgCl@SiO_2-4.1% photocatalyst.

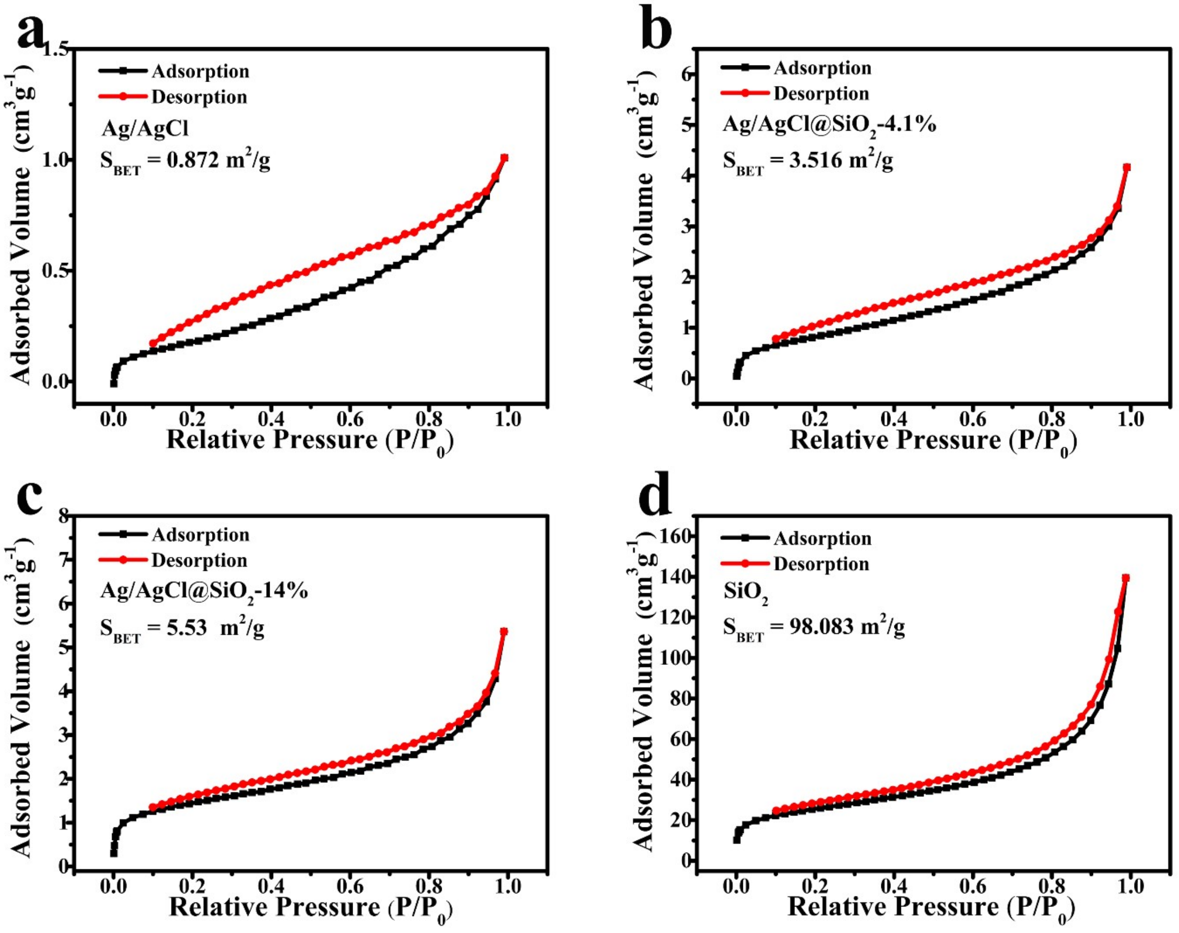

Fig. S6. N_2 **adsorption-desorption isotherms of Ag/AgCl@**SiO_2**-x.**

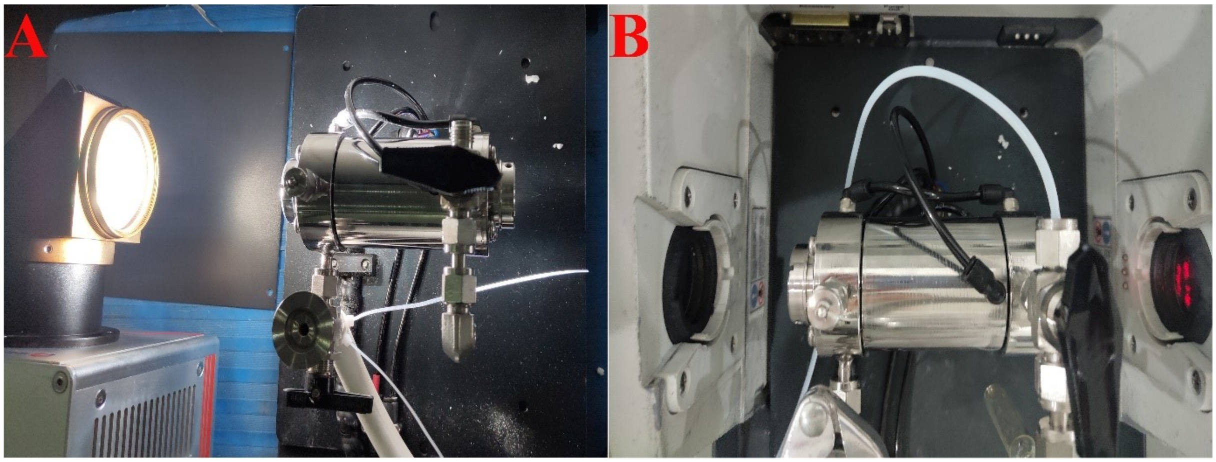

Fig. S7. Photograph of the semi in-situ IR analysis apparatus.

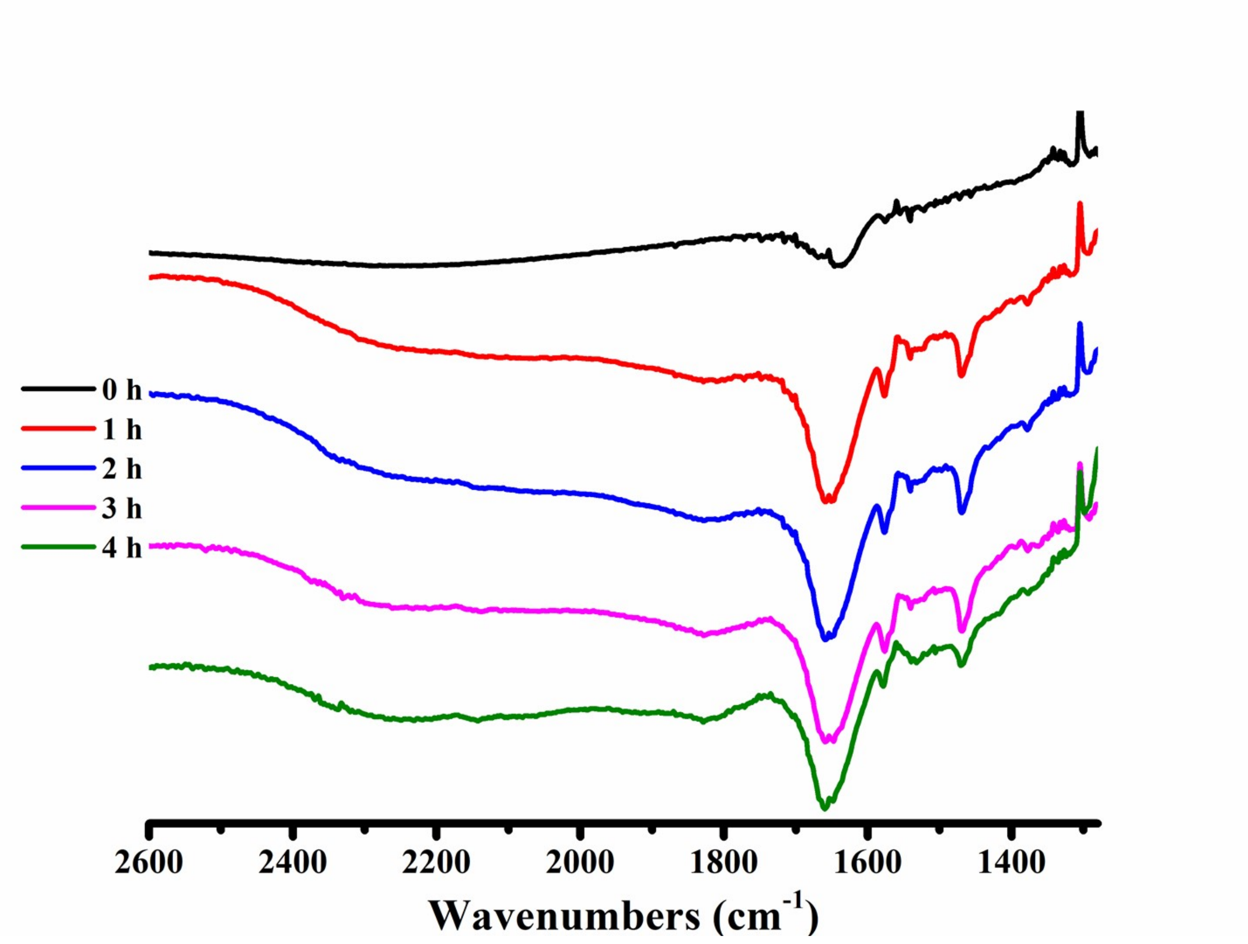

Fig. S8. FTIR spectra of gaseous phase during methane photocatalytic oxidation over the Ag/AgCl@SiO_2-4.1% catalyst as a function of reaction time.

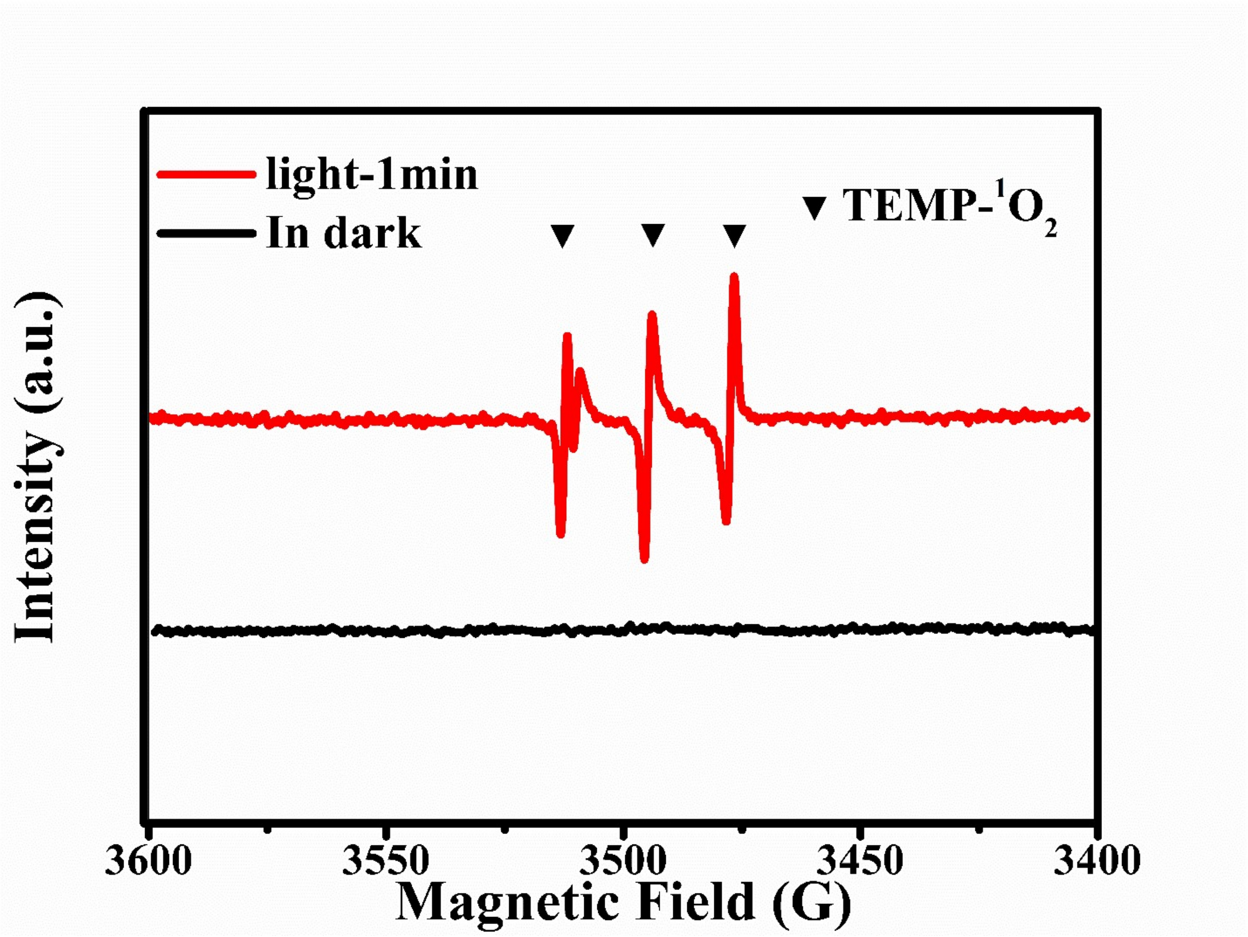

Fig. S9. In situ ESR spectra of Ag/AgCl@SiO_2-4.1% in the presence of TEMP

Table S1. ICP – OES analysis of Ag/AgCl@SiO_2-x.

Catalyst	The volume of TEOS addition (mL)	Si (wt%)
Ag/AgCl@SiO_2-1%	0.05	0.5
Ag/AgCl@SiO_2-4.1%	0.1	1.6
Ag/AgCl@SiO_2-9.6%	0.15	4.2
Ag/AgCl@SiO_2-14%	0.2	5.5
